# Supplementary material for: Label-Free Detection of DNA via Surface-Enhanced Raman Spectroscopy Using Au@Ag Nanoparticles
Source: Nanomaterials (Basel). 2022 Sep 8;12(18):3119. doi: 10.3390/nano12183119 (PMC9505376; doi:10.3390/nano12183119)
Supplement: Supplementary file 1 [file nanomaterials-12-03119-s001.zip › nanomaterials-1892474-supplementary.pdf]

Supplementary Materials

Label-Free Detection of DNA via Surface-Enhanced Raman Spectroscopy Using Au@Ag Nanoparticles

Ting Zhang [1,†], Xubin Quan [1,†], Naisi Cao [1], Zhaoying Zhang [2,*] and Yang Li [1,*]

[1] Department of Pharmaceutical Analysis and Analytical Chemistry, Research Center for Innovative Technology of Pharmaceutical Analysis, College of Pharmacy, Harbin Medical University, Harbin 150081, China

[2] The Fourth Hospital of Harbin Medical University, Harbin 150001, China

* Correspondence: zhzh_405@163.com (Z.Z.); liy@hrbmu.edu.cn (Y.L.).

† These authors contributed equally to this work.

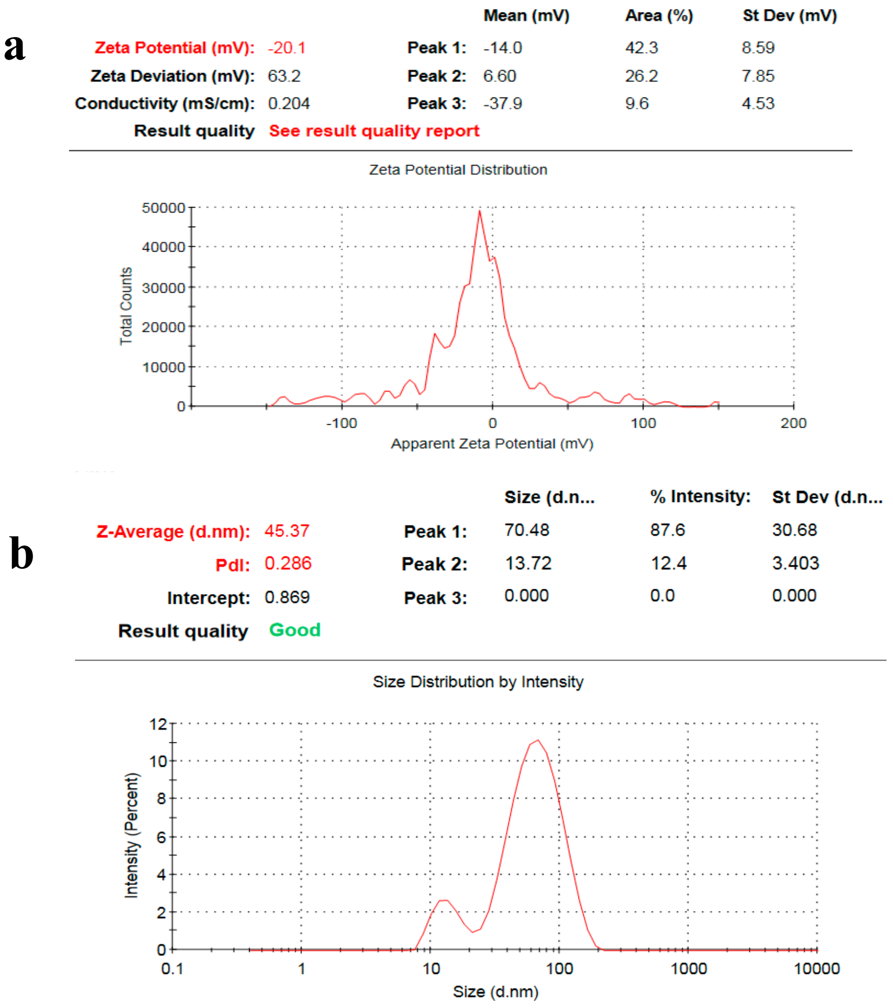

Figure S1. (**a**)The Zeta potential of Au@AgNPs; (**b**) The DLS data of Au@AgNPs.

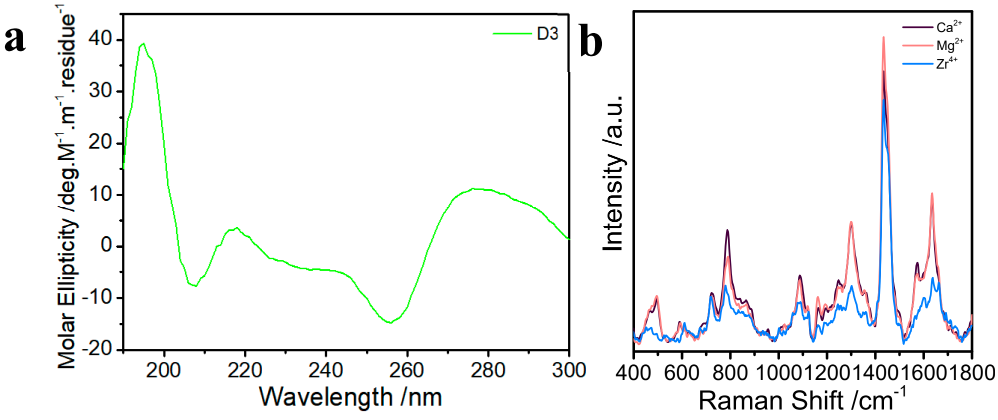

Figure S2. (**a**) CD spectra of the D3; (**b**) The D3 SERS spectrum of Au@AgICNPs (Brown line), Au@AgIMNPs (red line) and Au@AgIZNPs (blue line).

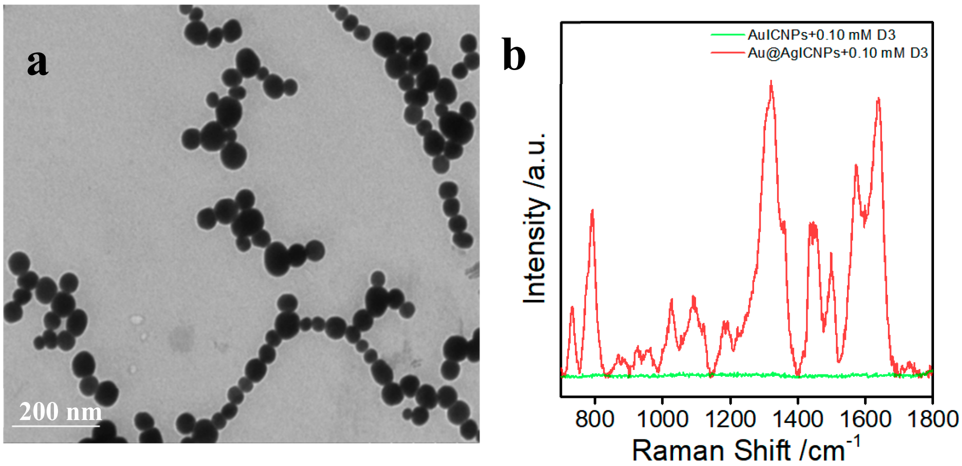

Figure S3. (**a**) The TEM of pure AuNPs; (**b**) The D3 SERS spectrum of AuICNPs with 0.1 mM D3 (green line) and Au@AgICNPs with 0.1 mM D3(red line).

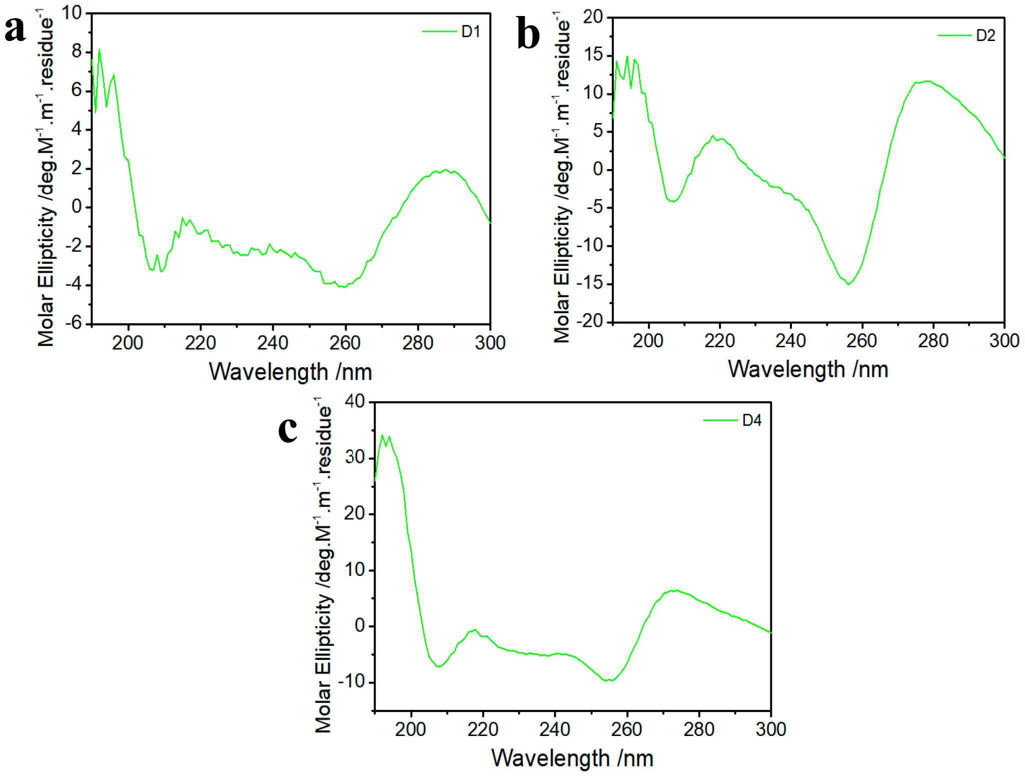

Figure S4. (**a**) The CD spectra of the D1; (**b**) The CD spectra of the D2; (**c**) The CD spectra of the D4.

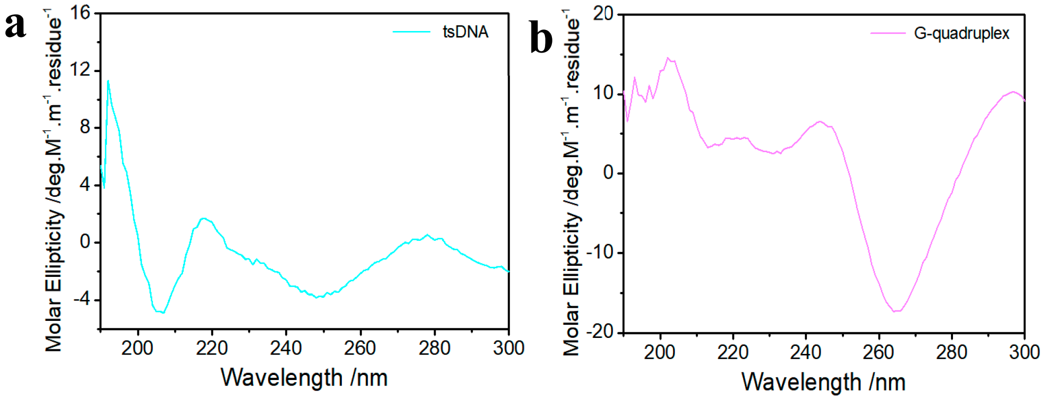

Figure S5. (**a**) The CD spectra of the tsDNA; (**b**) The CD spectra of the G-quadruplex.

Table S1. The characteristic peak positions of DNA of different structures.

Raman Shift		dsDNA	tsDNA
733 cm^{-1}	736 cm^{-1}	dA ring br	dA ring br
789 cm^{-1}	795 cm^{-1}	νs OPO; dC ring br	νs OPO; dT ring br
868 cm^{-1}	-	ν OPO, bk	-
962 cm^{-1}	-	d, dG δ R5	-
1028 cm^{-1}	1033 cm^{-1}	d (ν C-O)	d (ν C-O)
1093 cm^{-1}	1094 cm^{-1}	νs PO_2^-, bk	νs PO_2^-, bk
1323 cm^{-1}	1322 cm^{-1}	dG ν(C-N) δ(C8-H)	dG ν(C-N) δ(C8-H)
1360 cm^{-1}	-	dG C2'-endo/*syn*	-
1637 cm^{-1}	1632 cm^{-1}	dT, dC ν C5=C6	dT ν C5=C6

Raman Shift		dsDNA	G-quadurplex
733 cm^{-1}	713 cm^{-1}	dA ring br	dA ring br
789 cm^{-1}	789 cm^{-1}	νs OPO; dC ring br	νs OPO; dT ring br
868 cm^{-1}	-	ν OPO, bk	-
962 cm^{-1}	-	d, dG δ R5	-
1028 cm^{-1}	-	d (ν C-O)	-
1093 cm^{-1}	1087 cm^{-1}	νs PO_2^-, bk	νs PO_2^-, bk
-	1241 cm^{-1}	-	dT, dG δ NH(N2)
1323 cm^{-1}	-	dG ν(C-N) δ(C8-H)	-
1360 cm^{-1}	1358 cm^{-1}	dG C2'-endo/*syn*	dG ν(C-N) δ(C8-H) C2'-
1637 cm^{-1}	1644 cm^{-1}	dT, dC ν C5=C6	endo/*anti*
-	1764 cm^{-1}	-	dT ν C5=C6
			dG ν C=O (O6 interbase H-
			bond)

Note: bk=DNA - phosphate backbone, d =deoxyribose, ν=stretch, δ=deformation, br =breathe, s =symmetry.

Table S2. The motifs of tsDNA and G-quadurplex detected in the paper.

**tsDNA**	5′-AGA$_7$GGA-3′<br>5′-TCT$_7$CCT-3′
**G-quadurplex**	5′-G$_4$T$_4$G$_4$-3′
